# Constructing Tumor Cellular Cluster Models and Their Evolutionary Dynamics to Decode Tumor Evolution

**DOI:** 10.1002/cai2.70066

**Published:** 2026-06-01

**Authors:** Haili Qian, Fei Ma

**Affiliations:** ^1^ State Key Laboratory of Molecular Oncology, National Cancer Center/National Clinical Research Center for Cancer/Cancer Hospital Chinese Academy of Medical Sciences and Peking Union Medical College Beijing China; ^2^ Beijing Key Laboratory of Innovative Drug Research and Translational Study for Breast Cancer Beijing China; ^3^ Department of Medical Oncology, State Key Laboratory of Molecular Oncology, National Cancer Center/Cancer Hospital Chinese Academy of Medical Sciences and Peking Union Medical College Beijing China

1

Tumor research has undergone a transition from a cancer cell‐centered paradigm to a systematic evolutionary perspective. Cancer is a highly complex disease, and tumor initiation and progression have traditionally been interpreted primarily through genetic and molecular alterations within tumor cells. However, with the emergence of systems biology, this “cancer cell‐centered” view has gradually evolved into a “systems evolutionary perspective.” The most recent theoretical framework in oncology emphasizes that tumors are dynamic systems comprising multiple cell types and their interactions, highlighting that their evolution reflects system‐level remodeling driven by multidimensional signaling networks [1]. Within this framework, tumor cells, immune cells, stromal cells, and vascular‐associated cells form highly interconnected regulatory networks through molecular interactions and cell–cell communication. These networks collectively shape tumor initiation, progression, and therapeutic response. Consequently, approaches focusing solely on single molecules or individual cells are unable to sufficiently account for the complexity and dynamic behavior of tumors.

## 2 Cellular Clusters as Key Biological Mesoscale Structures Linking Cells and Tissues

In multiscale biological systems, cellular clusters—structural units positioned between single cells and tissues—have emerged as a critical level for understanding tumor complexity. These clusters can be broadly categorized into homogeneous and heterogeneous types based on their composition. Homogeneous clusters are mainly composed of phenotypically similar tumor cells and characterized by clonal expansion and localized proliferative advantage, serving as an important source of tumor genetic heterogeneity [2]. In contrast, heterogeneous clusters consist of tumor cells together with immune cells, fibroblasts, endothelial cells, and other cell types, forming functionally specialized microenvironmental structures [3]. These cellular clusters are not merely spatial aggregates but also functionally integrated systems shaped by molecular signaling networks and intercellular communication. Within such systems, chemokines, cytokines, and receptor‐mediated signaling establish multilevel feedback regulation, giving rise to “functional emergence” beyond the behavior of individual cells. Accordingly, tumor initiation and progression can be viewed as a continuous process of structural reorganization and functional emergence at the level of cellular clusters.

## 3 From Descriptive Data‐Driven Analysis to Rule‐Based Interpretation: The Need for New Theoretical and Dynamical Frameworks

In recent years, rapid advances in single‐cell sequencing and spatial multi‐omics technologies have brought tumor research into a high‐resolution data era. However, the large‐scale data sets generated by these approaches remain largely limited to descriptive and correlative analyses, with insufficient logic for systematic integration and dynamic modeling. Current research paradigms face several key challenges: (1) high‐dimensional data are difficult to reduce to interpretable low‐dimensional structures; (2) models often rely on empirical fitting without a unifying theoretical foundation; and (3) there is a lack of dynamical frameworks capable of capturing functional emergence and evolutionary patterns to biologically organized real cancer complexity. As a result, the understanding of tumor evolution remains fragmented despite the rapid growth of data and has yet to converge on a unified formulation comparable to the “fundamental laws” described in physics.

## 4 The Mathematical Nature of Biological Systems and the Computability of Tumor Evolution

Biological processes exhibit inherent mathematical properties, with underlying structures that can be described in formal mathematical terms. The success of the protein structure prediction model AlphaFold, together with the highly ordered dynamical trajectories observed during embryonic development, provides strong evidence for the mathematical nature and predictability of biological systems [4, 5, 6]. Tumors are an aberrantly evolving biological system, and their mathematic dynamics are characterized by shifts in system parameters and partial signal magnification, rather than an isolation from the fundamental mathematical framework of biology. Accordingly, the evolution of tumor cellular clusters can, in principle, be described by a mathematical logic that is amenable to data representation, mathematical modeling, and outcome prediction.

## 5 Mesoscience May Offer a Valuable Methodological Framework for Understanding Complex Biological Systems

When we attempt to directly apply microscopic principles to macroscopic systems, we often find that the inferences drawn from the micro‐level cannot be readily verified at the macro‐level, and biology is no exception. This reflects the reality that as we move from the micro‐ to the macro‐level, new functionality emerges. Mesoscience is a field that arose precisely from the need to understand the distinctive principles governing the intermediate realm between the “micro” and the “macro.” It holds that all complex systems are underpinned by a common logic of competition and coordination, thus giving rise to entirely new structures and behaviors when microscopic units come together [7, 8]. This principle has been widely validated in fields such as fluid dynamics, chemical engineering, and life sciences. Mesoscience also offers a valuable methodological framework for understanding complex biological systems. In tumor systems, cellular clusters represent a prototypical mesoscale structure, linking molecular and subcellular processes to tissue‐ and organ‐level functions. By incorporating mesoscale modeling, it becomes possible to integrate molecular signaling, cellular behavior, and population‐level organization within a unified framework, enabling analysis of cross‐scale dynamics. Recent studies have extended this framework to complex biological networks such as the immune system, further supporting its general relevance in life sciences [9, 10].

## 6 Mesoscience‐Based “Digital Cellular Clusters” Advancing Tumor Systems Biology and Precision Therapy

**Figure 1:**
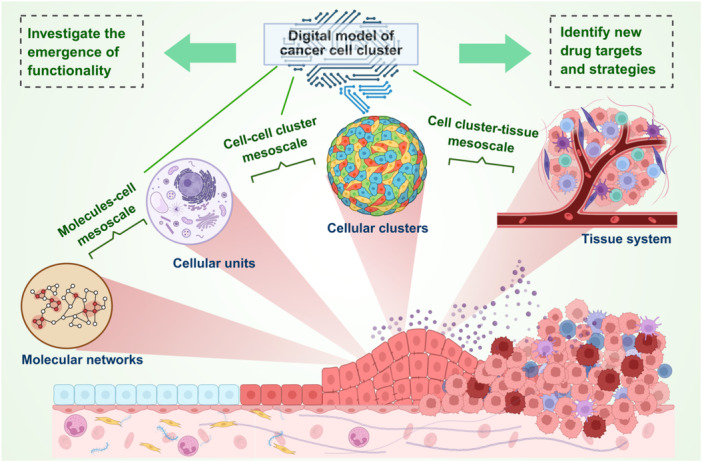
A schematic showing the mesoscale structure of tumor evolution. Created in BioRender. Fucong, Z. (2026). https://BioRender.com/9od1b53.

In response to the challenges in integrating data‐driven approaches with theoretical modeling, we propose the concept of “digital cellular clusters.” This concept is grounded in mesoscale structures and integrates molecular networks, subcellular functional modules, cellular states, and cellular cluster architectures into a multilevel‐coupled dynamical system. By constructing a hierarchical “molecule–subcellular–cell–cellular cluster” model, it becomes possible to dynamically simulate and quantitatively describe tumor evolution (Figure 1). The digital cellular clusters model emphasizes the faithful representation of biological structure and function, capturing key processes such as cell–cell communication, network regulation, and collective evolution. This framework not only facilitates the identification of mechanisms driving tumor progression but also enables the prediction of therapeutic response and resistance trajectories, thereby providing a theoretical basis for precision oncology.

## 7 Conclusion

Tumor research is undergoing a critical transition from data‐driven to rule‐based paradigms. Mesoscale modeling centered on cellular clusters offers a new avenue for uncovering the mathematical principles underlying tumor evolution. With the continued integration of multi‐omics data and dynamical modeling, the digital cellular cluster framework holds promise for achieving systematic biological decoding of tumor initiation and progression and predicting therapeutic responses. This emerging paradigm is expected not only to advance oncology from a descriptive to a computational discipline but also to serve as a key bridge between basic research and clinical application.

## Author Contributions

**Haili Qian:** conceptualization (equal), writing – original draft (equal), writing – review and editing (equal). **Fei Ma:** conceptualization (equal), writing – original draft (equal), writing – review and editing (equal).

## Ethics Statement

The authors have nothing to report.

## Consent

The authors have nothing to report.

## Conflicts of Interest

Professor Haili Qian and Professor Fei Ma are members of the *Cancer Innovation* Editorial Board. To minimize bias, they were excluded from all editorial decision‐making related to the acceptance of this article for publication.

## Data Availability

Data sharing is not applicable to this article as no data sets were generated or analyzed during the current study.

## References

[1] D. Hanahan , “Hallmarks of Cancer: Then and Now, and Beyond,” Cell 189, no. 8 (2026): 2254–2277, 10.1016/j.cell.2025.12.049. 41616779

[2] S. Migliozzi , B. Adabbo , L. Garofano , et al., “Restraint of Cancer Cell Plasticity by Spatial Homotypic Clustering,” Cancer Cell 43, no. 12 (2025): 2206–2223.e10, 10.1016/j.ccell.2025.08.009. 40972572 PMC12453603

[3] N. Aceto , “Bring Along Your Friends: Homotypic and Heterotypic Circulating Tumor Cell Clustering to Accelerate Metastasis,” Biomedical Journal 43, no. 1 (2020): 18–23, 10.1016/j.bj.2019.11.002. 32200952 PMC7090281

[4] M. G. Krokidis , D. E. Koumadorakis , K. Lazaros , et al., “AlphaFold3: An Overview of Applications and Performance Insights,” International Journal of Molecular Sciences 26, no. 8 (2025): 3671, 10.3390/ijms26083671. 40332289 PMC12027460

[5] L. Chen , Q. Li , K. F. A. Nasif , et al., “AI‐Driven Deep Learning Techniques in Protein Structure Prediction,” International Journal of Molecular Sciences 25, no. 15 (2024): 8426, 10.3390/ijms25158426. 39125995 PMC11313475

[6] D. Dirvanauskas , R. Maskeliunas , V. Raudonis , and R. Damasevicius , “Embryo Development Stage Prediction Algorithm for Automated Time Lapse Incubators,” Computer Methods and Programs in Biomedicine 177 (2019): 161–174, 10.1016/j.cmpb.2019.05.027. 31319944

[7] J. Li and W. Huang , “From Multiscale to Mesoscience: Addressing Mesoscales in Mesoregimes of Different Levels,” Annual Review of Chemical and Biomolecular Engineering 9 (2018): 41–60, 10.1146/annurev-chembioeng-060817-084249. 29553825

[8] J. Chen , Y. Ren , W.‐L. Huang , L. Zhang , and J. Li , “Multilevel Mesoscale Complexities in Mesoregimes: Challenges in Chemical and Biochemical Engineering,” Annual Review of Chemical and Biomolecular Engineering 13 (2022): 431–455, 10.1146/annurev-chembioeng-092220-115031. 35378042

[9] H. Qian and A. S. Beltran , “Mesoscience in Cell Biology and Cancer Research,” Cancer Innovation 1, no. 4 (2022): 271–284, 10.1002/cai2.33. 38089088 PMC10686186

[10] Y. Ren , A.‐G. Wu , Y. Shi , Y.‐F. Ping , J.‐H. Li , and X.‐W. Bian , “Challenges in the Immune System: Mesoscale and Mesoregime Complexity,” Cancer Innovation 4, no. 5 (2025): e70030, 10.1002/cai2.70030. 41122466 PMC12536266

